# Altered static and dynamic spontaneous brain activity in patients with dysthyroid optic neuropathy: a resting-state fMRI study

**DOI:** 10.3389/fnins.2024.1530967

**Published:** 2025-01-10

**Authors:** Jinling Lu, Hao Hu, Jiang Zhou, Wenhao Jiang, Xiongying Pu, Huanhuan Chen, Xiaoquan Xu, Feiyun Wu

**Affiliations:** ^1^Department of Radiology, The First Affiliated Hospital of Nanjing Medical University, Nanjing, China; ^2^Department of Endocrinology, The First Affiliated Hospital of Nanjing Medical University, Nanjing, China

**Keywords:** dysthyroid optic neuropathy, amplitude of low-frequency fluctuation, regional homogeneity, dynamic analysis, resting state fMRI

## Abstract

**Purpose:**

To investigate static and dynamic brain functional alterations in dysthyroid optic neuropathy (DON) using resting-state functional MRI (rs-fMRI) with the amplitude of low-frequency fluctuation (ALFF) and regional homogeneity (ReHo).

**Materials and methods:**

Fifty-seven thyroid-associated ophthalmopathy (TAO) patients (23 DON and 34 non-DON) and 27 healthy controls (HCs) underwent rs-fMRI scans. Static and dynamic ALFF (sALFF and dALFF) and ReHo (sReHo and dReHo) values were compared between groups. The support-vector machine (SVM) classification method was used to examine the diagnostic performance of the identified models.

**Results:**

Compared to non-DON patients, DON patients showed decreased sALFF in the bilateral lingual gyrus (LING) and right cuneus (CUN), alongside increased sALFF in the bilateral medial part of the superior frontal gyrus, right dorsolateral part of the superior frontal gyrus (SFGdor), and right precentral gyrus. DON patients also exhibited decreased dALFF in the left LING and right CUN, together with increased dALFF in the right orbital part of the middle frontal gyrus and right SFGdor in comparison to non-DON patients. Meanwhile, DON patients had lower sReHo in the right LING, and higher sReHo and dReHo in the right supramarginal gyrus compared to non-DON patients. When detecting DON, the dALFF model showed optimal diagnostic performance (AUC 0.9987).

**Conclusion:**

Dysthyroid optic neuropathy patients exhibited both static and dynamic brain functional alterations in visual, cognitive, and emotion-related brain regions, deepening our current understanding of the underlying neural mechanisms of this disease. Rs-fMRI-based metrics, especially dALFF, may serve as relevant neuroimaging markers for diagnosing DON.

## 1 Introduction

Dysthyroid optic neuropathy (DON) is one of the most serious complications of thyroid-associated ophthalmopathy (TAO) and has a frequently insidious onset (Wu et al., 2022; Neigel et al., 1988). The primary clinical manifestations of DON include decreased visual acuity, visual field defects, color vision dysfunction, and optic disk edema (McKeag et al., 2007). If untreated, permanent damage to visual function can occur. The pathogenesis of DON is not fully established, but optic nerve compression resulting from orbital apex crowding syndrome is a widely accepted mechanism (Wu et al., 2021). Many recent studies have found that there may be an association between visual dysfunction and altered brain function in DON patients (Liu et al., 2023; Jiang et al., 2021). DON patients have also been reported to be more prone to anxiety and depression than non-DON patients (Jiang et al., 2021), indicating that DON is not simply an ocular disease, but is also accompanied by neuropsychic disturbances. There is thus a need to investigate the underlying neurobiological mechanisms of DON, concurrently facilitating the accurate diagnosis and synergistic treatment of affected patients.

Resting-state functional MRI (rs-fMRI) is a non-invasive technology for exploring neuronal activity based on blood oxygen level-dependent (BOLD) signals (Biswal, 2012). Amplitude of low-frequency fluctuation (ALFF) and regional homogeneity (ReHo) are two widely used rs-fMRI metrics. ALFF reflects the regional intensity of spontaneous brain activity, while ReHo indicates the consistency of neuronal activity in the local brain area (Zang et al., 2007; Lv et al., 2019; Zang et al., 2004; Ji et al., 2020). Previously, several studies of TAO have uncovered altered ALFF and ReHo in brain areas connected with vision, emotion, and cognition, indicating a disturbance of localized brain activity (Chen et al., 2021; Jiang et al., 2023; Wu et al., 2024; Wen et al., 2023). With respect to the DON subgroup, one recent study reported ReHo abnormalities associated with visual dysfunction, suggesting that there may be a specific neural pattern associated with DON (Liu et al., 2023). It has also been observed that DON patients had decreased degree centrality (DC) values in the primary and secondary visual cortex compared to healthy controls (HCs) (Wu et al., 2023).

However, most previous investigations have only focused on the static changes in brain activity (Chen et al., 2021; Jiang et al., 2023; Wu et al., 2024; Wen et al., 2023). A growing body of evidence has indicated that brain activity fluctuates over time (Goldenberg and Galván, 2015; Wei et al., 2022; Calhoun et al., 2014). Therefore, to capture uncontrolled but recurring brain activity patterns effectively, a dynamic analysis based on a sliding window approach has been implemented (Ma et al., 2014). Extensive studies have confirmed that these dynamic properties are reliable with good reproducibility, allowing them to be of value in distinguishing between patients and HCs (Liao et al., 2018; Chen et al., 2021). Jiang et al. (2023) revealed that TAO patients exhibited functional alterations both in brain activity and connectivity using a dynamic analysis method based on rs-fMRI. However, the dynamic alterations of brain activity in DON patients remain largely unexplored.

Therefore, the purpose of this study was to investigate the changes in spontaneous brain activity in DON patients using both static and dynamic ALFF and ReHo. Moreover, we applied a support vector machine (SVM) method to determine whether aberrant ALFF and ReHo could distinguish DON patients from non-DON controls.

## 2 Materials and methods

### 2.1 Subjects

This prospective study was approved by our institutional review board. All subjects volunteered to participate in the study and signed an informed consent form before enrollment. A total of 23 DON patients (12 females and 11 males, mean age 56.04 ± 11.46 years) and 34 non-DON patients (24 females and 10 males, mean age 51.35 ± 7.07 years) were recruited from our hospital. At the same time, 27 HCs (17 females and 10 males, mean age 52.44 ± 7.93 years) were recruited from the local community. TAO was clinically diagnosed based on the Bartley criteria (Bartley and Gorman, 1995), and DON was diagnosed based on at least two of the following clinical presentations: (1) best-corrected visual acuity (BCVA) < 0.8; (2) visual field defects; (3) relative afferent pupillary defects; (4) impairment of color vision; and (5) optic disk edema. Inclusion criteria were as follows: (1) sufficient imaging quality for further analysis; (2) no other orbital pathologies; (3) no history of orbital surgery; (4) no history of nervous system disease or mental disease; and (5) good physical condition with the ability to complete the questionnaire tests. Exclusion criteria were as follows: (1) any evidence of other orbital diseases; (2) history of orbital surgery; (3) history of neurological or psychiatric disorders; (4) contraindications to MRI examination; or (5) history of radiotherapy or systemic glucocorticoid therapy within 3 months (Hu et al., 2024). The presence of anxiety and/or depression was not an exclusion criterion if TAO or DON was the primary clinical diagnosis.

### 2.2 Clinical assessment

Demographic and relevant clinical variables were acquired from participants. The scoring of disease activity was performed according to the modified 7-point clinical activity score (CAS) (Bartalena et al., 2021). The duration of TAO was defined from the onset of relevant ophthalmic symptoms to the date of the MRI scan. The visual field index (VFI) was obtained from a visual field test, and proptosis and BCVA tests were conducted. Serum levels of free triiodothyronine (FT3), free thyroxine (FT4), and thyrotropin (TSH) were also collected. Cognitive and psychometric testing were performed for each subject before MRI scanning. Anxiety and depressive degrees were assessed using the Hamilton Anxiety Rating Scale (HARS) and Hamilton Depression Rating Scale (HDRS), respectively. The Montreal Cognitive Assessment (MOCA) was used to evaluate cognitive function. In addition, a TAO-specific quality of life (QoL) questionnaire, focusing on visual function and appearance, was administered to all patients before they underwent MRI examinations (Lin et al., 2015).

### 2.3 MRI data acquisition

All rs-fMRI data were acquired using a 3.0-T MRI system (Magnetom Skyra; Siemens Healthcare, Erlangen, Germany) with a 20-channel head and neck coil. Earplugs and foam pads were employed to reduce scanner noise and minimize head motion. During the scan, subjects were instructed to lie still in the supine position with their eyes closed, remaining relaxed and awake. An MPRAGE sequence was used to acquire high-resolution sagittal structural T1-weighted images with the following parameters: repetition time (TR) = 1,900 ms, echo time (TE) = 2.45 ms, flip angle = 9°, field of view (FOV) = 256 × 256 mm^2^, matrix = 256 × 256, thickness = 1.0 mm, number of slices = 176, and voxel size =1 × 1 × 1 mm^3^. Functional images were then acquired axially by applying an echo planar imaging sequence. The specific parameters were as follows: TR = 2,000 ms, TE = 30 ms, flip angle = 90°, FOV = 240 × 240 mm^2^, matrix = 64 × 64, thickness = 4.0 mm, number of slices = 35 and voxel size = 3.75 × 3.75 × 4 mm^3^. The total scanning time was 12 min 26 s.

### 2.4 Data preprocessing

All the rs-fMRI data were preprocessed by applying the DPABI toolbox^1^ (Yan et al., 2016) based on Statistical Parametric Mapping (SPM) 12^2^. The first 10 images of each dataset were discarded to ensure magnetization stability and allow the participants to adapt to the scanning environment. Slice-timing and head motion correction were performed on the remaining 230 consecutive images. Participants would be excluded if the maximum value of the head rotation (translation) movement was over 2.5^°^ (2.5 mm). In order to reduce the impact of head motion, we calculated the mean frame-wise displacement (FD) (Power et al., 2012) and included it as a covariate in the group-level analysis. Then, the images were spatially normalized to the Montreal Neurological Institute (MNI) template (resampling voxel size = 3 × 3 × 3 mm^3^) using T1 image unified segmentation. Nuisance covariates, including the Friston 24-parameters of head motion, the brain’s global signal, and white matter and cerebrospinal fluid signals, were regressed out. A 6-mm full-width half-maximum (FWHM) Gaussian kernel was applied to smooth the images. However, for static and dynamic ReHo analyses, smoothing was performed after calculating the ReHo. Finally, the data were temporally filtered, using a frequency of 0.01–0.08 Hz to reduce the influence of low-frequency drift and high-frequency noise.

### 2.5 Static ALFF and static ReHo analyses

Static ALFF (sALFF) was calculated using DPABI software. Each voxel’s time series was transformed into a frequency domain by Fourier transform. The square root of the power spectrum was calculated and then averaged across the 0.01–0.08 Hz frequency range. The average square root was taken as the sALFF value for each voxel. To standardize the value, each voxel’s sALFF value was then divided by the sALFF values’ global mean (Zang et al., 2007). DPABI software was also used to compute static ReHo (sReHo), and Kendall’s Coefficient of Concordance was used to calculate the similarity between a given voxel and its adjacent 27 voxels (Zang et al., 2004). Then, the sReHo values of each voxel were divided by the global mean of sReHo values.

### 2.6 Dynamic ALFF and dynamic ReHo analyses

The analyses of dynamic ALFF (dALFF) and dynamic ReHo (dReHo) were carried out using the Temporal Dynamic Analysis (TDA) toolkits in DPABI, which is based on a sliding-window approach. To exclude spurious fluctuations, prior studies have demonstrated that the minimum window length should not be smaller than 1/fmin, where fmin is the minimum frequency of time series (Leonardi and Van De Ville, 2015). A window length of 50 TRs (100 s) was considered optimal for maintaining the balance between recording a fast-shifting dynamic relationship and producing reliable estimates of the inter-regional correlations (Cui et al., 2020). Hence, we chose 50 TRs (100 s) as the sliding window length and 1 TR (2 s) as the sliding step size. This process produced 181 windows for each subject. We obtained ALFF and ReHo maps from each sliding window and calculated the variance of ALFF and ReHo maps across windows to depict the time-varying overall brain activity; namely, the dALFF and dReHo maps. These maps were subsequently normalized to *z*-scores.

### 2.7 Statistical analyses

All demographic and clinical data were analyzed using SPSS (version 26, IBM SPSS Inc). Among the three groups, normally distributed continuous variables were compared with analysis of covariance (ANCOVA) tests, while non-normally distributed variables were compared with Kruskal–Wallis tests. Between the two groups, normally distributed continuous variables were compared using Student’s *t*-tests, while non-normally distributed variables were compared using Mann–Whitney *U*-tests. The *χ*^2^ tests were applied for categorical variables. The threshold for statistical significance was set at *p* < 0.05 (two-tailed).

For the imaging data, including sALFF, sReHo, dALFF, and dReHo values, ANCOVAs were first conducted to compare differences among the three groups within a gray-matter mask with age, gender, education, and mean FD as covariates. Subsequently, brain regions with significant differences were extracted as a mask. Two-sample *post hoc t*-tests were then performed between any two groups. Gaussian Random Field (GRF) correction with a voxel-level threshold of *p* < 0.001 and a cluster-level threshold of *p* < 0.05 (two-tailed) was used for multiple comparison correction. BrainNet Viewer was used to visualize alterations of brain models. In the DON group, Pearson’s correlation analyses were conducted to assess the relationships between the rs-fMRI metrics in significant brain regions and the normally distributed clinical parameters. And Spearman’s correlation analyses were performed to evaluate the relationships between the rs-fMRI metrics in significant brain regions and ranked clinical data and data with non-normal distribution.

### 2.8 SVM analyses

The SVM is a supervised machine learning approach that has been widely used in the diagnosis of neurological disorders (Orrù et al., 2012). We performed SVM analyses using LIBSVM software (Chang et al., 2000) to further detect whether the rs-fMRI values of significant clusters could be diagnostic biomarkers to identify DON patients. Four rs-fMRI models were built: the (1) sALFF; (2) dALFF; (3) sReHo; and (4) dReHo models. A leave-one-out cross-validation (LOOCV) strategy was employed to verify the classification performance due to the limited number of samples (Zhu et al., 2021; Pereira et al., 2009). Receiver operating characteristic (ROC) curves were computed to evaluate the classification ability of the SVM models. DeLong’s test was applied to compare diagnostic efficiencies between models. The significance of classification accuracy was measured using the non-parametric permutation test with 5,000 permutations.

### 2.9 Validation analyses

To confirm our findings regarding dALFF and dReHo alterations, an additional two different sliding-window lengths [30 TR (60 s) and 70 TR (140 s)] were used to perform further validation analyses.

## 3 Results

### 3.1 Demographic and clinical characteristics

The demographic and clinical data of all participants are listed in Table 1. There were no significant differences in age (*p* = 0.134), gender (*p* = 0.368), years of education (*p* = 0.134), or smoking history (*p* = 0.455) among the DON, non-DON, and HC groups. Both the DON and non-DON groups showed significantly lower MoCA scores and lower BCVA (all *p* < 0.001) compared to HCs, together with higher HDRS and HARS scores (both *p* < 0.05). DON patients showed significantly lower QoL scores related to visual function, MoCA scores, and BCVA, as well as higher HDRS scores, CAS, and proptosis relative to the non-DON group (all *p* < 0.05). There was no significant difference in QoL scores associated with appearance; TAO duration; serum levels of FT3, FT4, and TSH; or HARS scores between the DON and non-DON groups.

**Table 1:** Demographic and clinical characteristics of participants.

Items	DON (*n* = 23)	Non-DON (*n* = 34)	HC (*n* = 27)	*p*
Age (years)	56.04 ± 11.46	51.35 ± 7.07	52.44 ± 7.93	0.134
Sex (male/female)	11/12	10/24	10/17	0.368
Education level (years)	9.87 ± 3.86	11.15 ± 3.18	10.19 ± 2.80	0.134
Handedness (right/left)	23/0	34/0	27/0	>0.999
TAO duration (months)	12.04 ± 9.59	14.56 ± 14.49		0.948
Smoking history (yes/no)	9/14	11/23	13/14	0.455
CAS (OD)	4.48 ± 1.27	2.21 ± 1.23		<0.001
CAS (OS)	4.39 ± 1.31	2.24 ± 1.23		<0.001
FT3 (pmol/L)	5.12 ± 1.38	4.93 ± 1.17		0.579
FT4 (pmol/L)	16.72 ± 5.67	17.01 ± 4.14		0.474
TSH (mlU/L)	2.58 ± 7.28	1.62 ± 1.62		0.199
BCVA (OD)	0.60 ± 0.28	0.99 ± 0.13	1.00 ± 0.12	<0.001
BCVA (OS)	0.60 ± 0.27	0.97 ± 0.14	1.00 ± 0.11	<0.001
VFI (OD)	0.73 ± 0.31			
VFI (OS)	0.82 ± 0.30			
Proptosis (OD) (mm)	20.67 ± 3.22	17.35 ± 2.79		<0.001
Proptosis (OS) (mm)	21.35 ± 3.53	17.62 ± 3.03		<0.001
Total score of QoL				
Visual functioning	43.48 ± 18.01	67.33 ± 25.75		<0.001
Appearance	59.24 ± 20.80	66.18 ± 17.21		0.176
Total score of HDRS	14.48 ± 5.27	9.32 ± 5.02	2.78 ± 2.08	<0.001
Total score of HARS	15.26 ± 4.67	12.68 ± 5.54	3.19 ± 1.71	<0.001
Total score of MoCA	23.19 ± 1.71	26.00 ± 3.55	29.04 ± 1.02	<0.001

Data are presented as mean ± standard deviation.

BCVA, best-corrected visual acuity; CAS, clinical activity score; DON, dysthyroid optic neuropathy; FT3, free triiodothyronine; FT4, free thyroxine; HARS, Hamilton Anxiety Rating Scale; HC, healthy control; HDRS, Hamilton Depression Rating Scale; MD, mean deviation; MoCA, Montreal Cognitive Assessment; OD, oculus dexter; OS, oculus sinister; PSD, pattern SD; QoL, quality of life; TAO, thyroid-associated ophthalmopathy; TSH, thyrotropin; VFI, visual field index.

### 3.2 Static and dynamic ALFF values among groups

**Figure 1:**
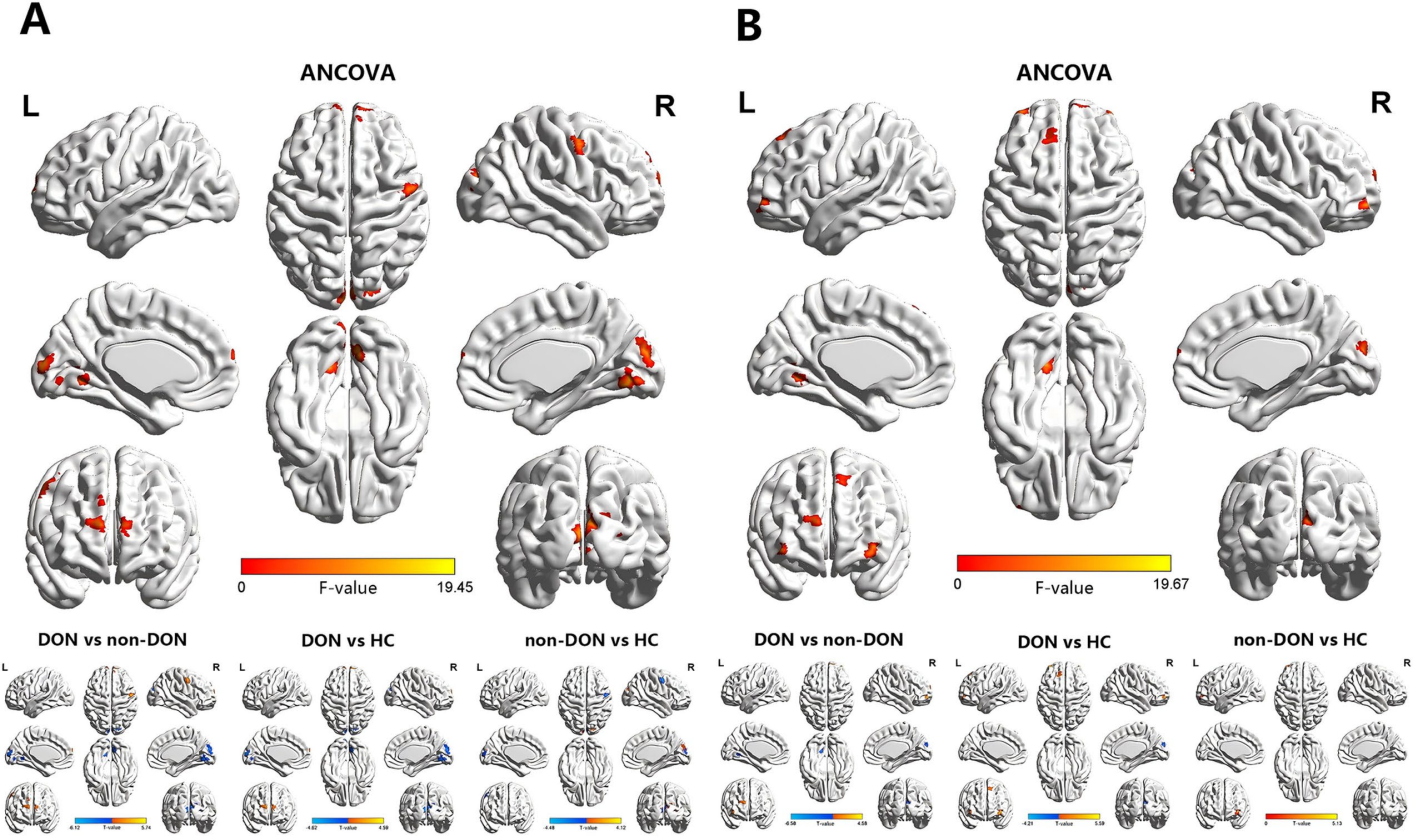
Brain regions exhibiting significant differences in sALFF **(A)** and dALFF **(B)** values among the DON, non-DON, and HC groups (voxel level: *p* < 0.001, cluster level: *p* < 0.05, GRF corrected). In the ANCOVA results, red denotes regions that differed among the three groups. In the post-hoc pairwise comparison results, warm and cold colors indicate increased and decreased sALFF and dALFF values, respectively. ANCOVA, analysis of covariance; sALFF, static amplitude of low-frequency fluctuation; dALFF, dynamic amplitude of low-frequency fluctuation; DON, dysthyroid optic neuropathy; HC, healthy controls; R, right; L, left.

The ANCOVA results showed significant sALFF differences among the three groups, primarily in the bilateral lingual gyrus (LING), right cuneus (CUN), left medial part of the superior frontal gyrus (SFGmed), right dorsolateral part of the superior frontal gyrus (SFGdor) and right precentral gyrus (PreCG) (Supplementary Table S1 and Figure 1). In the pairwise comparisons, the DON group exhibited decreased sALFF in the bilateral LING and right CUN, alongside increased sALFF in the bilateral SFGmed, right SFGdor and right PreCG compared to the non-DON group. As compared to HCs, the DON group exhibited decreased sALFF in the right LING and right CUN, as well as increased sALFF in the right SFGmed and left SFGdor. In addition, non-DON patients displayed lower sALFF in the right PreCG but higher sALFF in the right superior occipital gyrus (SOG) compared to HCs (Table 2 and Figure 1).

**Table 2:** Brain areas showing significant differences in sALFF and dALFF values across the three groups.

Conditions	Brain region	BA	Cluster size	MNI coordinates			*T*-values
				*X*	*Y*	*Z*	
sALFF							
DON vs. non-DON	LING.L	18/19	22	-15	-60	0	−4.735
	LING.R	17/18	31	9	−72	−9	−5.0474
	CUN.R	17/18/19	94	12	−90	18	−6.1229
	SFGmed.L	10	18	−12	69	15	5.1497
	SFGmed.R	10	15	9	69	18	5.0404
	SFGdor.R	9	14	27	60	30	4.3897
	PreCG.R	6	14	51	−3	54	5.743
DON vs. HCs	LING.R	17/18	7	12	−72	−3	−3.9176
	CUN.R	18/19	33	−6	−96	9	−4.6234
	SFGdor.L	10	10	−12	69	18	4.1951
	SFGmed.R	9/10	11	12	69	21	4.5897
Non-DON vs. HCs	PreCG.R	4/6	14	54	−3	51	−4.4839
	SOG.R	17/18/19	9	21	−87	15	4.118
dALFF							
DON vs. non-DON	LING.L	17/18/19	21	−15	−63	0	−5.1789
	CUN.R	18/19	21	12	−87	18	−6.5771
	ORBmid.R	10/46/47	8	42	57	−6	4.3227
	SFGdor.R	10	10	21	66	24	4.585
DON vs. HCs	CUN.R	18	11	9	−90	18	−4.2082
	SFGdor.L	8/9	6	−15	42	54	5.586
	ORBmid.L	10/46/47	9	−36	60	−6	4.7966
	ORBmid.R	10/46/47	9	42	57	−6	4.2965
Non-DON vs. HCs	ORBmid.L	10/46/47	8	−36	60	−3	5.1287

GRF correction, voxel level: *p* < 0.001, cluster level: *p* < 0.05.

DON, dysthyroid optic neuropathy; HC, healthy controls; BA, Brodmann’s areas; MNI, Montreal Neurological Institute; sALFF, static amplitude of low-frequency fluctuation; dALFF, dynamic amplitude of low-frequency fluctuation; R, right; L, left; LING, lingual gyrus; CUN, cuneus; SFGmed, medial part of superior frontal gyrus; SFGdor, dorsolateral part of superior frontal gyrus; PreCG, precentral gyrus; SOG, superior occipital gyrus; ORBmid, orbital part of middle frontal gyrus.

In terms of the dALFF analysis, the ANCOVA results showed significant dALFF differences among the three groups, primarily in the left LING, right CUN, bilateral orbital part of the middle frontal gyrus (ORBmid), right SFGmed and left SFGdor (Supplementary Table S1 and Figure 1). In the pairwise comparisons, the DON group exhibited decreased dALFF in the left LING and right CUN, but increased dALFF in the right SFGdor and right ORBmid compared to non-DONs. When compared with HCs, the DON group showed decreased dALFF in the right CUN, as well as increased dALFF in the left SFGdor and bilateral ORBmid. Moreover, the non-DON group showed higher dALFF in the left ORBmid compared to HCs (Table 2 and Figure 1).

### 3.3 Static and dynamic ReHo values among groups

**Figure 2:**
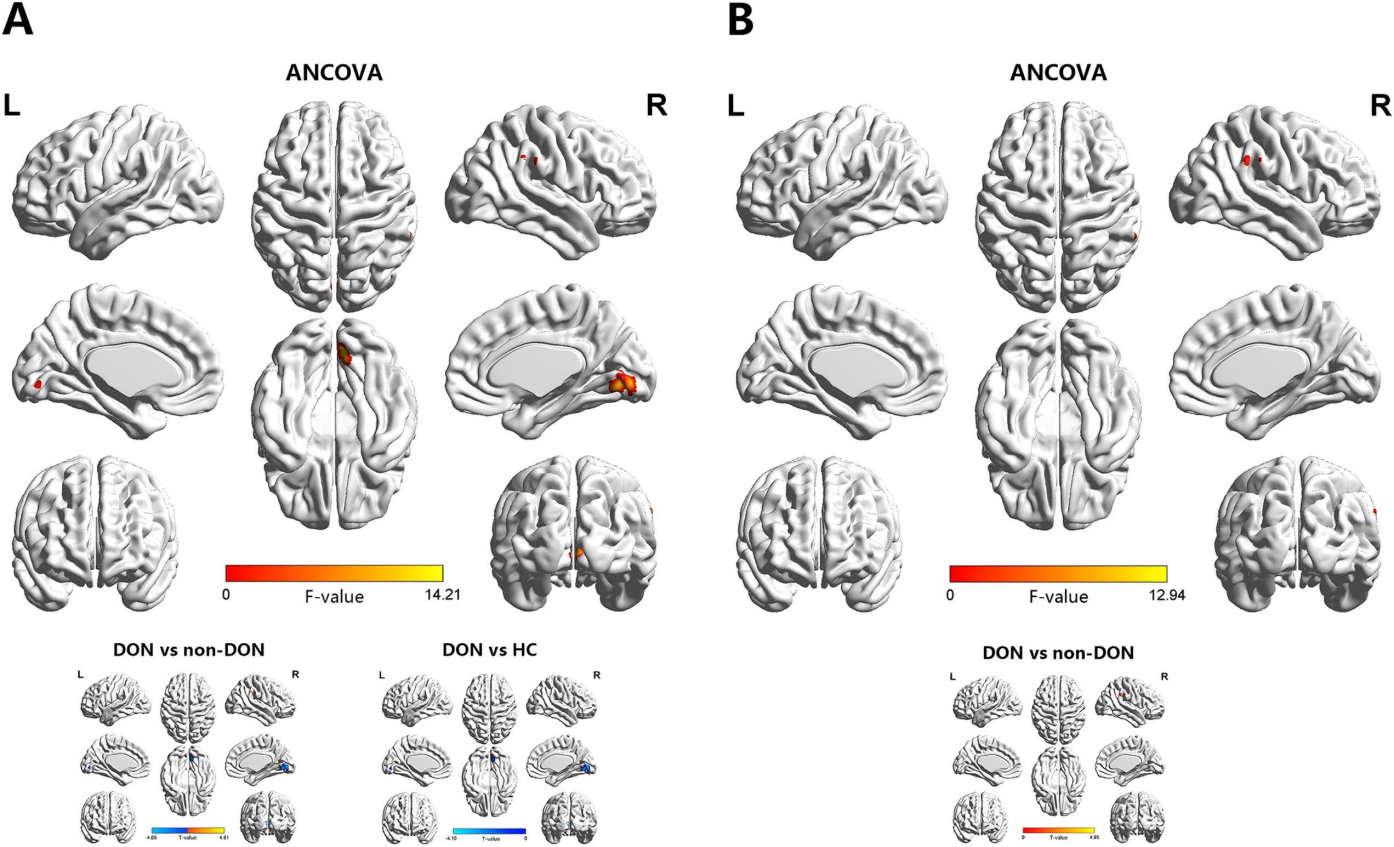
Brain regions showing significant differences in sReHo **(A)** and dReHo **(B)** values among the DON, non-DON, and HC groups (voxel level: *p* < 0.001, cluster level: *p* < 0.05, GRF corrected). In the ANCOVA results, red denotes regions that differed among the three groups. In the post-hoc pairwise comparison results, warm and cold colors indicate increased and decreased sReHo and dReHo values, respectively. ANCOVA, analysis of covariance; sReHo, static regional homogeneity; dReHo, dynamic regional homogeneity; DON, dysthyroid optic neuropathy; HC, healthy controls; R, right; L, left.

The ANCOVA results showed significant sReHo differences among the three groups, primarily in the right LING and right supramarginal gyrus (SMG) (Supplementary Table S1 and Figure 2). In the pairwise comparisons, compared with the non-DON group, the DON group exhibited decreased sReHo in the right LING, as well as increased sReHo in the right SMG. When compared with HCs, the DON group showed decreased sReHo in the right calcarine fissure and surrounding cortex (CAL) (Table 3 and Figure 2).

**Table 3:** Brain areas showing significant differences in sReHo and dReHo values across the three groups.

Conditions	Brain region	BA	Cluster size	MNI coordinates			*T*-values
				*X*	*Y*	*Z*	
sReHo							
DON vs. non-DON	LING.R	17/18	53	9	−72	−6	−4.6472
	SMG.R	40	24	69	−36	39	4.8063
DON vs. HCs	CAL.R	17/18	19	9	−75	3	−4.1028
dReHo							
DON vs. non-DON	SMG.R	40	18	66	−36	39	4.952

GRF correction, voxel level: *p* < 0.001, cluster level: *p* < 0.05.

DON, dysthyroid optic neuropathy; HC, healthy controls; BA, Brodmann’s areas; MNI, Montreal Neurological Institute; sReHo, static regional homogeneity; dReHo, dynamic regional homogeneity; R, right; LING, lingual gyrus; SMG, supramarginal gyrus; CAL, calcarine fissure and surrounding cortex.

In terms of the dReHo analysis, the ANCOVA results showed significant dReHo differences among the three groups in the right SMG (Supplementary Table S1 and Figure 2). In the pairwise comparisons, the DON group also exhibited increased dReHo in the right SMG compared with the non-DON group (Table 3 and Figure 2).

### 3.4 Correlation analysis

**Figure 3:**
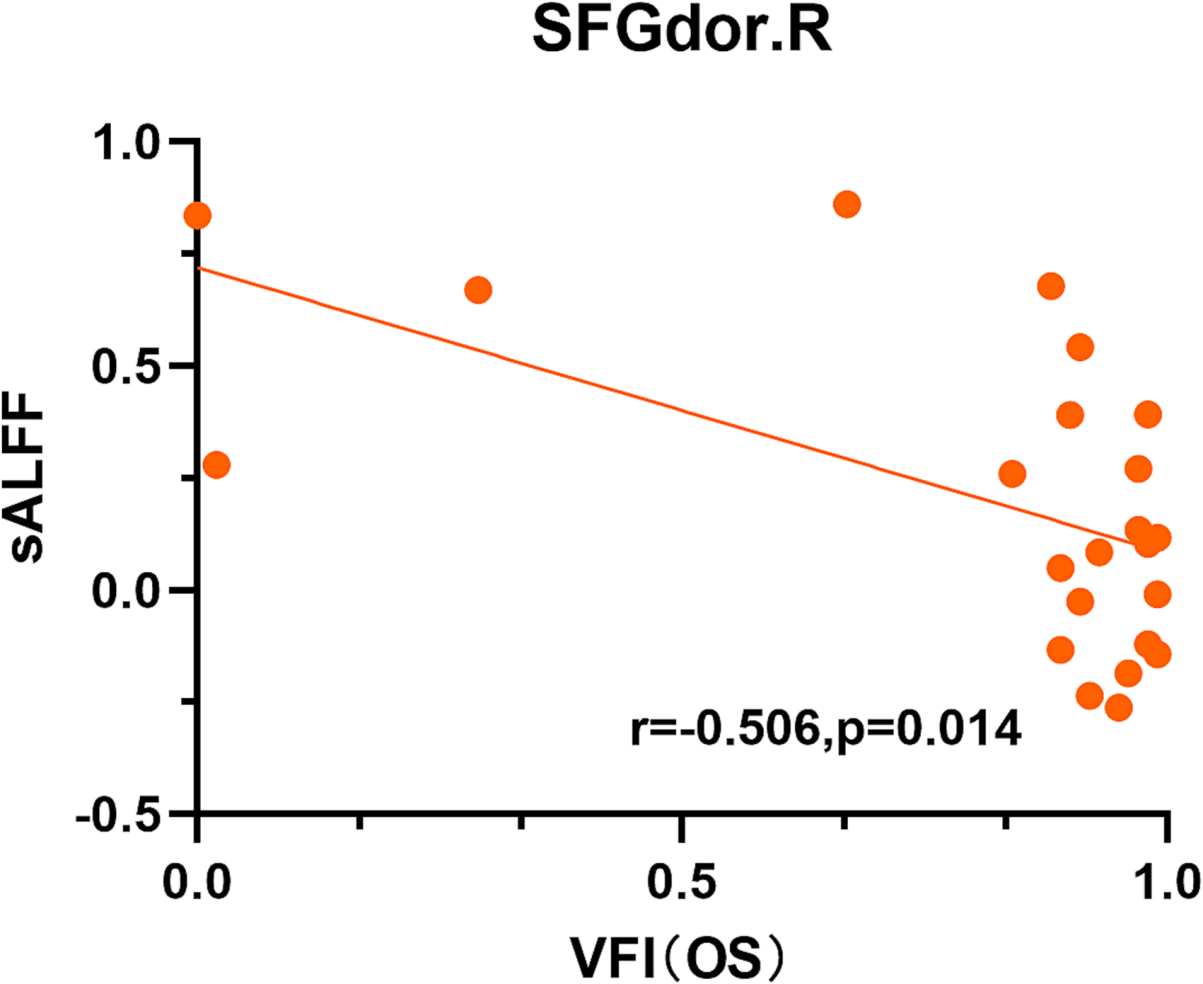
Scatter diagrams showing significant correlations between clinical characteristics and the sALFF values in DON patients. sALFF in the right SFGdor was negatively correlated with the VFI of the left eye (Spearman’s, *r* = −0.506, *p* = 0.014), namely, the higher VFI corresponds to a lower sALFF. sALFF, static amplitude of low-frequency fluctuation; SFGdor, dorsolateral part of superior frontal gyrus, VFI, visual field index; OS, oculus sinister.

The correlation analysis results are shown in Figure 3. In the DON cohort, sALFF in the right SFGdor was negatively correlated with the VFI of the left eye (Spearman’s, *r* = −0.506, *p* = 0.014) (Figure 3). No significant correlations were found between rs-fMRI metrics and other clinical measures.

### 3.5 SVM classification results

**Figure 4:**
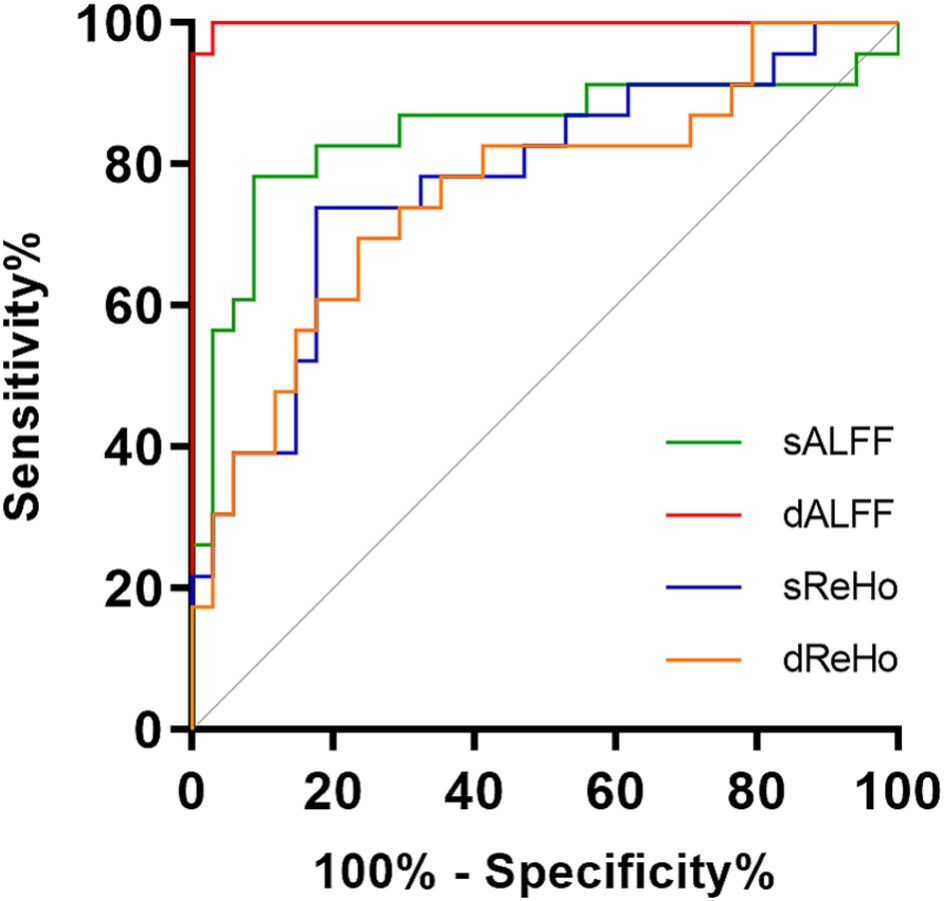
Receiver operating characteristic curves of the SVM-based classifiers. SVM, support vector machine; sALFF, static amplitude of low-frequency fluctuation; dALFF, dynamic amplitude of low-frequency fluctuation; sReHo, static regional homogeneity; dReHo, dynamic regional homogeneity.

The dALFF model exhibited the best diagnostic performance (AUC 0.9987, accuracy 98.25%, sensitivity 100.00%, specificity 97.06%), followed by the sALFF model (AUC 0.8440, accuracy 85.96%, sensitivity 78.26%, specificity 91.18%), sReHo model (AUC 0.7762, accuracy 71.93%, sensitivity 73.91%, specificity 82.35%), and dReHo model (AUC 0.7609, accuracy 70.18%, sensitivity 69.57%, specificity 76.47%). The diagnostic efficiency of the dALFF model was significantly higher than that of the other SVM models (*p* = 0.0155, 0.0007, 0.0004, respectively). ROC curves for different rs-fMRI models are shown in Figure 4.

### 3.6 Validation results

Analyses using different sliding-window lengths (30 TRs and 70 TRs) supported our main findings of dALFF alterations (Supplementary Table S2 and Supplementary Figure S1).

## 4 Discussion

In this study, we applied both static and dynamic ALFF and ReHo analyses to investigate brain functional alterations in DON patients. Our study had two main findings. First, DON patients exhibited decreased ALFFs and ReHos in the visual cortex, together with increased ALFFs and ReHos in cognitive and emotion-related brain regions compared to the non-DON patients. Second, the machine learning models developed based on the identified rs-fMRI metrics achieved good performance when distinguishing between DON patients and non-DON patients. Among the tested models, the dALFF model showed the optimal classification performance, highlighting the significance of dynamic analysis. These findings deepen our current understanding of the neurobiological mechanisms of DON, providing new insights that can aid the accurate diagnosis of this disease.

DON patients exhibited significantly decreased ALFF value and its temporal variability in the LING and CUN compared to both non-DON patients and HCs. Additionally, sReHo was lower in the LING in DON patients than in non-DON patients and it was also lower in the CAL in DON patients relative to HCs. These brain regions mentioned above are located in the occipital lobe, mainly involving Brodmann areas (BA) 17, 18, and 19. The occipital lobe is the visual cortical center and is involved in visual information processing (Qin et al., 2020). Specifically, the LING is involved in the encoding of visual memories (Roland and Gulyás, 1995), while the CAL belongs to the primary visual cortex (V1), which is the first target for visual input within the neocortex (Resulaj, 2021). The CUN has been reported to be connected with spatial orientation and reflexive movement of the eyes, thereby contributing to the stabilization of retinal imaging (Lan et al., 2019; Schraa-Tam et al., 2009). Previous rs-fMRI studies focused on other ocular disorders, including diabetic retinopathy, primary angle-closure glaucoma, blindness, and amblyopia have consistently verified the disruptions in brain activity in the occipital lobe (Huang et al., 2021; Fu et al., 2022; Huang et al., 2019; Dai et al., 2019). Wu et al. (2023) reported significantly decreased DC values in the BA 17 and 18 areas in DON patients. Therefore, considering the significantly lower bilateral BCVA observed in DON patients, we deduced that the observed abnormal spontaneous neural activity with the altered temporal variability in the occipital lobe may reflect damage to visual function in these patients.

In this study, we also observed that DON patients had significantly increased ALFF value and its temporal variability in the SFGdor, SFGmed, and ORBmid relative to non-DON patients and HCs. DON patients also had increased sALFF in the PreCG compared to non-DON patients. Additionally, ReHo value and its temporal variability were found to be higher in the SMG in DON patients relative to non-DON patients. The SFGmed, SFGdor, and ORBmid are important components of the prefrontal cortex (PFC), which is thought to be associated with various cognitive functions, including working memory, executive functions, and decision-making (Arnsten, 2015; Carlén, 2017). Previous rs-fMRI studies have discovered enhanced PFC brain activity in Alzheimer’s disease and mild cognitive impairment patients (Zhou et al., 2020; Cai et al., 2018); this may be attributable to a compensatory response to cognitive impairment. Hence, we speculated that the increased ALFF value and its temporal variability in the PFC of DON patients may similarly occur as a compensatory mechanism, given that cognitive scores were also reduced in individuals with DON. Curiously, sALFF in the right SFGdor of DON patients was found to be negatively correlated with the VFI of the left eye. Given that the PFC also partly participates in integrating visual information (Zhang et al., 2023), this enhanced brain activity may also reflect a possible compensatory change in visual function. The PreCG plays a role in the execution of movement (Grefkes et al., 2008) and is located in the primary motor cortex. Our finding suggested that the executive control network of DON patients may also be impaired.

Notably, the SFGmed and SFGdor are also important components of the DMN, which is related to emotional regulation (Zi et al., 2022). The DMN sustains baseline brain activity, and it has been widely reported to exhibit functional abnormalities in psychiatric disorders, particularly depression (Liu and Huang, 2020; Sudheimer et al., 2015). Chen et al. (2023) demonstrated that dysfunction of the DMN is correlated with concomitant depression and anxiety in TAO. Moreover, the SMG is a component of the ventral attention network (VAN), which is engaged in the bottom-up stimulus-driven attentional modulation (Corbetta et al., 2008). Recent research on TAO has revealed that VAN abnormalities may be linked to negative affective states. Therefore, in light of the higher total scores of HDRS and HARS in DON patients, we posited that the functional alterations in the DMN and VAN in the current patient cohort may also be associated with patients’ emotional disturbances.

Through an SVM-based machine learning strategy, we observed that dALFF union was the best-performing diagnostic model, with an AUC of 0.9987, while those of other unions were significantly lower (AUC, 0.7609–0.8440). In recent years, dynamic brain functional properties have attracted increasing attention (Preti et al., 2017; Ma et al., 2020; Ma et al., 2021). Dynamic metrics have been certified as reliable indicators, reflecting the temporal variability of brain activity relative to static metrics (Liao et al., 2018; Ma et al., 2020). Higher brain signal flexibility is believed to correspond to more effective processes, which lead to the greater stability of behavioral patterns (Garrett et al., 2013; Kielar et al., 2016). Previous rs-fMRI studies of TAO and other diseases have demonstrated that dALFF and dReHo are able to effectively differentiate patients from HCs, and can offer a more complete perspective on the neurobiological mechanisms of these diseases (Jiang et al., 2023; Ma et al., 2021; Tian et al., 2023; Tian et al., 2021; Wu et al., 2023). In the current study, the observed results of dALFF and dReHo reflected the disturbance of temporal variability of brain activity in DON patients, potentially contributing to a further understanding of the underlying neurobiological pattern of DON. The identified performance of dALFF union highlight the prospects for the application of dynamic analyses of brain activity in the diagnosis of DON, thereby facilitating the precise assessment and treatment of the disease.

Our study has several limitations. First, the sample size was relatively small such that future studies with a larger sample size are needed to validate the findings. Second, the cross-sectional design of our study may limit the assessment of potential brain alterations associated with disease progression. Longitudinal prospective analyses in future research should be conducted to track the changes in dynamic brain activity over time in patients with DON. Last, future research utilizing functional connectivity analyses of rs-fMRI as well as multi-model neuroimaging would facilitate the full understanding of the neural pattern of DON.

In conclusion, our study revealed that DON patients exhibit altered static and dynamic brain activity in cortices associated with vision, cognition, and emotion, reflecting corresponding functional impairments. These findings enhance our understanding of the neurobiological mechanisms of DON while also providing us with potential neural imaging biomarkers of the disease.

## Data Availability

The raw data supporting the conclusions of this article will be made available by the authors, without undue reservation.

## Footnotes

^1^ http://restfmri.net/DPABI

^2^ http://www.fil.ion.ucl.ac.uk/spm/
