# Supplementary material for: Altered static and dynamic spontaneous brain activity in patients with dysthyroid optic neuropathy: a resting-state fMRI study
Source: Front Neurosci. 2025 Jan 10;18:1530967. doi: 10.3389/fnins.2024.1530967 (PMC11757300; doi:10.3389/fnins.2024.1530967)
Supplement: Supplementary file 1 [file Data_Sheet_1.docx]

**Supplementary Materials**

**Table S1.** Brain areas showing significant differences in sALFF, dALFF, sReHo and dReHo values across the three groups.

| **Conditions** | **Brain region** | **BA** | **Cluster** | **MNI coordinates** | | | ***F* values** |
| --- | --- | --- | --- | --- | --- | --- | --- |
|  |  |  | **size** | **X** | **Y** | **Z** |  |
| **sALFF** | LING.L | 18/19 | 22 | -15 | -63 | 0 | 13.6199 |
|  | LING.R | 17/18 | 32 | 9 | -72 | -6 | 13.5742 |
|  | CUN.R | 17/18/19 | 99 | 15 | -87 | 15 | 19.4501 |
|  | SFGmed.L | 10 | 22 | -12 | 69 | 18 | 13.8124 |
|  | SFGdor.R | 9 | 38 | 9 | 69 | 18 | 18.373 |
|  | PreCG.R | 6 | 19 | 51 | -3 | 54 | 14.6893 |
| **dALFF** | LING.L | 17/18/19 | 24 | -15 | -63 | 0 | 15.5031 |
|  | CUN.R | 18/19 | 22 | 12 | -87 | 18 | 19.6718 |
|  | ORBmid.L | 10/46/47 | 11 | -36 | 60 | -3 | 11.8012 |
|  | ORBmid.R | 10/46/47 | 11 | 42 | 57 | -6 | 14.6863 |
|  | SFGmed.R | 10 | 13 | 15 | 66 | 21 | 14.8153 |
|  | SFGdor.L | 8/9 | 10 | -12 | 42 | 54 | 14.2275 |
| **sReHo** | LING.R | 17/18 | 56 | 9 | -72 | 0 | 12.5171 |
|  | SMG.R | 40 | 24 | 69 | -36 | 39 | 14.2076 |
| **dReHo** | SMG.R | 40 | 18 | 66 | -36 | 39 | 12.9402 |

Note: GRF correction, voxel level: *p* < 0.001, cluster level: *p* < 0.05

Abbreviations: BA, Brodmann's areas; MNI, Montreal Neurological Institute; sALFF, static amplitude of low-frequency fluctuation; dALFF, dynamic amplitude of low-frequency fluctuation; sReHo, static regional homogeneity; dReHo, dynamic regional homogeneity; R, right; L, left; LING, lingual gyrus; CUN, cuneus; SFGmed, medial part of superior frontal gyrus; SFGdor, dorsolateral part of superior frontal gyrus; PreCG, precentral gyrus; ORBmid, orbital part of middle frontal gyrus; SMG, supramarginal gyrus.

**Table S2.** Brain areas showing significant differences in dALFF values across the three groups with different sliding-window lengths (30 TRs and 70 TRs).

| **Conditions** | **Brain region** | **BA** | **Cluster** | **MNI coordinates** | | | ***T* values** |
| --- | --- | --- | --- | --- | --- | --- | --- |
|  |  |  | **size** | **X** | **Y** | **Z** |  |
| **30 TRs** |  |  |  |  |  |  |  |
| DON vs non-DON | LING.L | 17/18/19 | 30 | -15 | -63 | 0 | -5.8292 |
|  | LING.R | 17/18 | 10 | 9 | -72 | -6 | -4.4617 |
|  | CUN.R | 17/18 | 36 | 12 | -87 | 18 | -6.7647 |
|  | SFGmed.L | 10 | 18 | -12 | 69 | 18 | 4.9195 |
|  | SFGmed.R | 10 | 17 | 21 | 66 | 24 | 4.5107 |
| DON vs HCs | LING.R | 17/18 | 9 | 9 | -72 | -3 | -3.9896 |
|  | CUN.R | 18 | 19 | 6 | -90 | 18 | -4.5831 |
|  | SFGdor.R | 10 | 11 | 12 | 66 | 21 | 4.4977 |
| **70 TRs** |  |  |  |  |  |  |  |
| DON vs non-DON | LING.L | 17/18 | 13 | -12 | -66 | 3 | -4.3019 |
|  | CUN.R | 18/19 | 15 | 12 | -87 | 18 | -5.6811 |
|  | SFGdor.R | 10 | 7 | 21 | 66 | 24 | 4.2291 |
|  | ORBmid.R | 10/46 | 8 | 42 | 57 | -6 | 4.4592 |
| DON vs HCs | ORBmid.R | 10/46/47 | 9 | 42 | 60 | -6 | 4.615 |
|  | SFGdor.L | 8/9 | 6 | -15 | 42 | 54 | 5.2824 |

Note: GRF correction, voxel level: *p* < 0.001, cluster level: *p* < 0.05

Abbreviations: DON, dysthyroid optic neuropathy; HC, healthy controls; BA, Brodmann's areas; MNI, Montreal Neurological Institute; dALFF, dynamic amplitude of low-frequency fluctuation; R, right; L, left; LING, lingual gyrus; CUN, cuneus; SFGmed, medial part of superior frontal gyrus; SFGdor, dorsolateral part of superior frontal gyrus; ORBmid, orbital part of middle frontal gyrus.

**Figure S1.** Brain areas showing significant differences in dALFF values among DON, non-DON and HC groups with different sliding-window lengths (30 TRs and 70 TRs) (voxel level: *p* < 0.001, cluster level: *p* < 0.05, GRF corrected). In the ANCOVA results, red denotes regions that differed among the three groups. In the post-hoc pairwise comparison results, warm and cold colors indicate increased and decreased dALFF values, respectively. ANCOVA, analysis of covariance; dALFF, dynamic amplitude of low-frequency fluctuation; DON, dysthyroid optic neuropathy; HC, healthy controls; R, right; L, left.
